# Novel PCR-Based Technology for the Detection of Sunflower in Edible and Used Cooking Oils

**DOI:** 10.3390/foods13233760

**Published:** 2024-11-24

**Authors:** Tamara Kutateladze, Kakha Karchkhadze, Kakha Bitskinashvili, Boris Vishnepolsky, Tata Ninidze, David Mikeladze, Nelly Datukishvili

**Affiliations:** 1 Ivane Beritashvili Center of Experimental Biomedicine, 14 Gotua Str., Tbilisi 0160, Georgia; kutateladzet@yahoo.com (T.K.); kakha.bitskinashvili.1@iliauni.edu.ge (K.B.); b.vishnepolsky@gmail.com (B.V.); tata.ninidze.1@iliauni.edu.ge (T.N.); davit_mikeladze@iliauni.edu.ge (D.M.); 2 School of Natural Sciences and Medicine, Ilia State University, 3/5 Kakutsa Cholokashvili Ave., Tbilisi 0162, Georgia; kakhak@iliauni.edu.ge

**Keywords:** sunflower detection, PCR technology, genomic DNA extraction, edible oil, used cooking oil

## Abstract

Reliable detection of sunflower (*Helianthus annuus*) in edible and used cooking oil (UCO) is crucial for the sustainable production of food and biodiesel. In this study, a variety of sunflower oils (crude, cold pressed, extra virgin, refined, and UCO) were examined using different methods of DNA extraction and PCR amplification to develop an efficient technology for the identification of sunflower in oils. DNA extraction kits such as NucleoSpin Food, DNeasy mericon Food, and Olive Oil DNA Isolation as well as modified CTAB method were found to be able to isolate amplifiable genomic DNA from highly processed oils. Novel uniplex, double, and nested PCR systems targeting the sunflower-specific *helianthinin* gene were developed for efficient identification of sunflower. New sunflower DNA markers were revealed by uniplex PCRs. The combination of modified CTAB and nested PCR was demonstrated as a reliable, rapid, and cost-effective technology for detecting traces of sunflower in 700 μL of highly processed oil, including refined and used cooking oil. The study will contribute to both the food industry and the energy sector as developed methods can be used for oil authenticity testing in food and biodiesel production.

## 1. Introduction

Sunflower (*Helianthus annuus* L.) is one of the major oil crops in the world. It is widely cultivated in various countries and ranks third among oilseeds and fourth among vegetable oils in global production. Sunflower belongs to both oil and protein species due to its high content of oil (about 44%) and protein (about 16%). It is a rich source of healthy nutrients, minerals, antioxidants, and vitamins [1,2,3,4]. Therefore, sunflower is often utilized as a common ingredient in many processed food products such as butter, granola, cereals, breads, bakery products, trail mix, pasta, etc. In addition, sunflower ingredients may be present in processed foods due to contamination, which is a concern. However, sunflower belongs to food allergens. Ingestion of sunflower food by sensitized individuals can trigger a variety of symptoms ranging from mild to severe. Moreover, sunflowers might elicit life-threatening anaphylactic reactions [5,6,7,8]. Accurate information on the presence of sunflower in food can prevent health problems for consumers who are sensitive to sunflower.

It should be noted that sunflowers are mainly distributed for their edible oil, the content and quality of which is the most desirable. Sunflower oil is used in salads and cooking foods or can be hardened to make margarine. In addition, sunflower oil has been found as an undeclared additive in occasionally contaminated and adulterated oils [9,10]. Thus tracking sunflower in highly processed foods and oils is particularly important for safe food production.

Edible oils form a significant part of the food system and are used widely in human nutrition. They can provide essential fatty acids and vitamins. Oils are commonly used in industrial food production and home cooking worldwide. Edible oils are produced from oilseed crops such as soybean, rapeseed, sunflower, palm, corn, etc. In addition, there is a wide variety of blended oils [11]. Oils from different plants vary in quality, toxicity, health benefits, and price. The modern food industry faces a big challenge regarding the authenticity and adulteration of oils. Oil Contamination or fraudulent alteration is the oil industry’s biggest problem. Cross-contamination of oils can occur accidentally during the manufacture of oils. In addition, low-cost oils are intentionally mixed with expensive oils to increase profits in the oil business [9,10,12]. The significant health aspect is the different allergenicity of oilseeds. Allergenic oil crops can cause allergic disease in susceptible individuals. The allergenic proteins are mostly degraded during oil production, although available data indicate that even trace amounts of allergenic ingredients may trigger allergic reactions in sensitized persons [13,14].

In recent years, attention to the authenticity of edible oils has increased due to the emergence of recycled used cooking oils (UCO) in edible oils. UCO or WCO (waste cooking oils) is collected from the food industry and includes restaurant fryer oil, residential cooking oil, and blended oils. They contain toxic and dangerous compounds like aflatoxin, benzopyrene, etc. [15,16,17,18]. Thus, the presence of UCO in edible oil poses a significant threat to consumer health. To defend food safety and consumer-free choice, international regulations require monitoring and labeling of food ingredients. Thus, checking the authenticity of oil is a growing demand to protect laws and human health.

Today one of the most widespread and commercially viable raw materials for biodiesel production is UCO. Biodiesel is considered one of the most effective, renewable, environmentally friendly alternative biofuels. Biodiesel production has been steadily increasing, especially in the 21st century [19]. It has been reported that 95% of global biodiesel is produced from various edible oils, including UCO (10%). In recent years, more attention has been paid to using UCO due to the scarcity of edible oils, which are important for the food industry [20]. The UCO comprises different plant oils used in the food industry. The edible oils have different characteristics, such as types and ratios of the fatty acids, density, flash point, and kinematics viscosity which ultimately affect the quality of biodiesel made from these oils as well as the process of transesterification which is the most common method for biodiesel production in the industry. Therefore, the production of biodiesel from plant oils needs good planning and careful analysis of the raw materials, i.e., UCO and it is crucial to determine the composition of UCO before starting the biodiesel production process [20,21,22].

Sunflower ranks second (13%) among single edible oils in terms of global biodiesel production [20]. Due to the widespread sunflower cooking oil, it is one of the main components of UCO, therefore, quick and reliable analysis of UCO on the detection of sunflower oil is very important for planning the production of biodiesel, namely, adjusting the catalysts for the transesterification process which can determine the smooth, high quality and economically viable process for biodiesel production, thus being very important for the industry. Based on the described above reliable and efficient detection of sunflower in any oil is crucial for safe and high-quality food production as well as for biodiesel production.

To ensure the authenticity of the oil, it is necessary to verify the identity of the oily ingredients. To date, various approaches have been developed to determine the authenticity of edible oils. Analytical methods of detection often include nuclear magnetic resonance spectroscopy, high-performance liquid chromatography, gas chromatography, and mass spectrometry [9,23,24,25,26]. These methods are based on edible oils’ physical and chemical properties and analyze the content of various components such as fatty acids, tocopherols, amino acids, and sterols. However, data analysis in these methods is complicated because the chemical composition of edible oil depends on cultivars, seasons, and growing areas. Moreover, the detection limits of these methods are not sufficient to ensure the authenticity of the edible oils [27,28,29,30].

Molecular methods include DNA and protein-based techniques. DNA-based polymerase chain reaction (PCR) technology has been demonstrated as a promising tool for verifying the authenticity and traceability of processed foods. It has an advantage over protein-based methods because DNAs are more stable molecules than proteins in food processing. PCR allows accurate identification of species through their specific DNA sequences and does not depend on cultivars or environmental conditions. In recent years, PCR methods have been successfully used for the identification of oilseeds and the traceability of edible oils [12,29,30,31,32,33,34,35,36,37,38].

Despite existing methods, there are still significant challenges in effective oil authenticity detection due to the diversity of oilseeds and edible oils, DNA degradation, and small amounts of DNA in the oil. Available data indicate that oil identification methods depend on the texture, chemical and molecular properties, and characteristics of the oilseed [30]. Thus, the novel application of a DNA-based approach to the detection of each species of oilseeds is of particular interest.

A few works previously described PCR-based detection of sunflower in processed foods [39,40,41] and edible oils [10,30]. However, a method for identifying sunflowers in UCO has not yet been reported. Due to the demands of the food and biodiesel industry, there is a need for more sensitive and efficient detection of sunflower in oils.

In this study, a comprehensive investigation of sunflower oils (crude, cold pressed, extra virgin, refined, and UCO) was carried out using different methods of DNA extraction and PCR amplification. A rapid and inexpensive CTAB protocol was developed for the efficient extraction of genomic DNA from oils. Uniplex PCR systems targeting the sunflower-specific *helianthinin* gene allow the identification of new specific DNA markers. An efficient nested PCR method was developed and optimized for accurate and rapid detection of sunflower in both edible and used cooking oil.

## 2. Materials and Methods

### 2.1. Plant and Oil Materials

Sunflower (*Helianthus annuus* L.), soybean (*Glycine max*), and maize (*Zea mays*) seeds, as well as cold-pressed sunflower oil, were obtained from the local market of Tbilisi (Georgia). The seeds were milled with an electric grinder (Siemens, Munich, Germany) to obtain a homogenous fine flour.

The used cooking oil (UCO) was supplied by the Georgian biodiesel production company “Biodiesel Georgia LLC” (Tbilisi, Georgia), while crude sunflower oil was provided by the local company “AgroPro Ltd. (Tbilisi, Georgia)”. In addition, extra virgin and refined sunflower oils, as well as refined soybean and maize oils were purchased from supermarkets. All oils were stored in a refrigerator (4 °C) until analysis.

### 2.2. DNA Extraction

In the present work, four commercial kits and two variations of cetyltrimethylammonium bromide (CTAB)-based methods were used for DNA extraction. Genomic DNAs were isolated and purified from 100 mg of sunflower, maize, and soybean flour by DNeasy plant mini kit (Qiagen, Hilden, Germany). Moreover, two CTAB methods were used to isolate DNA from sunflower flour.

The oil samples were extracted with three commercial kits: NucleoSpin Food Kit (MACHEREY-NAGEL, Düren, Nordrhein-Westfalen, Germany), DNeasy mericon Food Kit (Qiagen, Hilden, Germany), Olive Oil DNA Isolation Kit (Norgen Biotek Corp., Thorold, OT, Canada) according to the manufacture instructions. In addition, two CTAB protocols were used for oil DNA extraction. The first standard CTAB method was described previously [42], while the second modified CTAB protocol was developed in this study and it is described below.

### 2.3. Pre-Concentration of the Oils

The oil was shaken vigorously before sampling. Different volumes of oil samples were tested. Oil samples were pre-concentrated before the extraction except for 700 µL of oil which was directly used. Two oil concentration protocols were used. The first method (pre-concentration I) was used to concentrate 24 mL, 48 mL, 150 mL, and 300 mL of oils and was performed by centrifugation in 25 mL tubes at 18,000× *g* for 30 min at 4 °C using a centrifuge Sigma 3-16PK (Sigma-Aldrich, Merck, Darmstadt, Germany) as previously described by Costa et al. [31].

The second protocol (pre-concentration II) was used to concentrate 700 μL, 12 mL, 24 mL, and 48 mL oil samples and was developed in this study. Pre-concentration protocol II involved several centrifugations of oil samples in 2 mL reaction tubes at 16,000× *g* for 20 min at room temperature using a MiniSpin plus centrifuge (Eppendorf, Hamburg, Germany). The amount of centrifugation depended on the final volume of the analyzed oil, considering that a maximum of twelve 2 mL test tubes were placed in the centrifuge. After each centrifugation, the upper portion of the oil was discarded, and the bottom portions (approximately 300 µL) were collected in two 2 mL tubes and centrifuged again at 16,000× *g* for 20 min. The supernatant was discarded and the oils remaining in the bottom (approximately 300–500 μL) were subjected to DNA extraction. It is worth noting that pellets were observed only in crude and cold-pressed oil samples after centrifugation.

### 2.4. Modified CTAB Method

Each 400 µL pre-concentrated oil sample was mixed with 500 µL of CTAB buffer (20 g CTAB/l, 1.4 M NaCl, 0.1 M Tris-HCl, 20 mM EDTA) and 20 µL of proteinase K (20 mg/mL). After the incubation at 65 °C for 1 h, with occasional stirring, 1 mL of chloroform was added and the mixture was centrifuged at 16,000× *g* for 15 min at room temperature. The upper phase was transferred to a new test tube and extracted again with 1 mL chloroform. After centrifugation at 16,000× *g* for 15 min, the aqueous phase was mixed with 0.6 volume parts of isopropanol (pre-cooled to −20 °C) and incubated at −20 °C overnight. Subsequently, after centrifugation at 16,000× *g* for 20 min, the supernatant was discarded and the pellet was washed with 500 µL of pre-chilled (at −20 °C) ethanol solution (70% *v*/*v*). After centrifugation, the supernatant was carefully discarded, the pellet was dried, and the DNA was dissolved in 50–100 µL of TE buffer. The extractions were done in duplicate assays for each sample. The concentration and purity of the extracted DNAs were analysed by NanoDrop One Microvolume UV-Vis Spectrophotometer (Thermo Fisher Scientific, Waltham, MA, USA).

### 2.5. Bioinformatic Analysis and Design of Oligonucleotide Primers

Based on the available literature and published GenBank databases (https://www.ncbi.nlm.nih.gov/nucleotide, accessed on 5 September 2023) the DNA sequence of sunflower 11S storage protein G3-D1 (helianthinin) (GenBank acc.no. M28832.1) was selected as a sunflower-specific gene [39]. Primer-BLAST [43] and PrimerQuest tools (https://eu.idtdna.com/PrimerQuest, accessed on 9 October 2023) were used to design oligonucleotide primers, and primer pairs for nested PCR systems targeting the sunflower *helianthinin* gene. The selected primers were screened for specificity using Primer-BLAST against various databases and plant species, including soybean and maize. No other suitable PCR products were identified for species other than sunflowers. The possible formation of dimers and secondary structures was also evaluated by FastPCR—version 3.6.18. [44]. In addition, primers targeting the eukaryotic 18S ribosomal RNA gene were taken from previous publications [45,46]. The PCR primers used in this study are shown in Table 1. All of the primers were synthesized and purified by Integrated DNA Technologies (Coralville, IA, USA). 

### 2.6. PCR Analysis

PCR amplification was performed in 25 µL total reaction volume containing 1 µL or 2 µL of DNA extract from seeds or oils, respectively, 1× Taq buffer, 1.5 mM MgCl_2_, 0.2 mM of each dNTP (Deoxynucleotide solution mix), 1.25 U Taq DNA polymerase (New England BioLabs, Ipswich, MA, USA), 0.5 μM of each primer. However, the concentration of primers 8S140 and 18S167 was 0.4 μM. In addition, nested and dual PCRs were performed using 2 μL of the 1st PCR mixture as a DNA template for the 2nd PCR. It should be noted that sterile water was used as a negative control in all PCR assays.

The PCR amplifications were carried out on the thermal cycler Techne TC-412 (Techne, Minneapolis, MN, USA) using the following program: preincubation at 95 °C for 4 min, 35 cycles consisting of DNA denaturation at 95 °C for 40 s, primer annealing at 56 °C for 45 s, elongation at 72 °C for 45 s; final extension step at 72 °C for 5 min. However, the number of cycles was 40 in the uniplex PCRs with primers heli188 and heli162 for the DNA samples extracted with the CTAB method from the concentrated oils.

The samples were tested at least in duplicate in each of the DNA extraction and PCR analyses in three independent experiments. The comparison of the outcomes revealed the reproducibility of the results.

### 2.7. Agarose Gel Electrophoresis

Both the genomic DNAs and PCR products were evaluated by electrophoresis (VWR International, Radnor, PA, USA) using 1.0% and 2.0% of agarose gels (SeaKem LE agarose; Cambrex, East Rutherford, NJ, USA) for genomic and amplified DNA, respectively. The agarose gels containing 1 μg/mL of ethidium bromide (Sigma-Aldrich, St. Louis, MO, USA) were visualized under ultraviolet (UV) light and a digital image was obtained using a gel documentation system PhotoDoc- It imaging system (UVP, Upland, CA, USA).

### 2.8. Statistical Analysis

DNA concentration and purity ratios (260/280 and 260/230) were analysed across different extraction methods and sample types using a two-way ANOVA to assess the effects of each factor and their interaction. Tukey’s Honestly Significant Difference (HSD) test was applied to identify statistically significant differences among groups, with a significance level of *p* < 0.05. Distinct group labels were assigned to significantly different groups. All statistical analyses were performed in R (version 4.4.1).

## 3. Results

### 3.1. Selection of Effective PCR Primers for Identification of Sunflower

The Helianthinins are the major group of storage proteins presenting only in sunflower (*Helianthus annuus*) seeds. They belong to the 11S globulin family. Thus, identification of the *Helianthinin* gene in DNA samples indicates the existence of sunflower [30,39,47].

**Figure 1:**
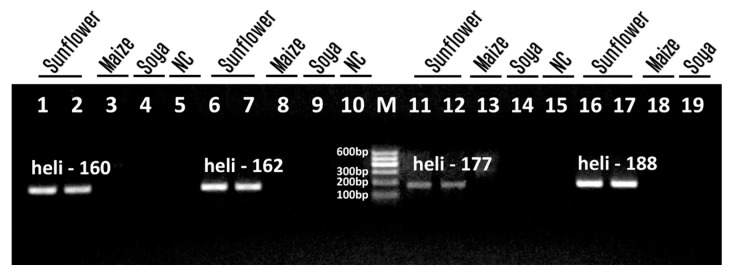
PCR detection of sunflower *Helianthinin* gene using primer pair heli160f/heli160r (lanes 1–5), heli162f/heli162r (lanes 6–10), heli177f/heli177r (lanes 11–15) and heli188f/heli188r (lanes 16–19). Samples: sunflower seeds (lanes 1–2, 6–7, 11–12, 16–17); maize seeds (lanes 3, 8, 13, 18); soybean seeds (lanes 4, 9, 14, 19); water-NC-negative control (lanes 5, 10, 15).

In the present work, new primers were designed targeting *Helianthinin* gene foreseen two requirements: (1) suitability for the nested PCR system and (2) yielding amplicon sizes less than 200 bp to overcome the problem of oil DNA degradation (Table 1). The efficiency of newly designed primer pairs targeting the *Helianthinin* gene was tested separately by uniplex PCRs (Figure 1). The genomic DNA extracted by the standard CTAB method from ground sunflower seeds was used in the amplification reaction. In addition, genomic DNAs from seeds of soybean and maize were applied to check the specificity of the PCR assay.

Figure 1 shows an agarose gel electrophoresis of the PCR products obtained by four primer pairs targeting the sunflower *Helianthinin* gene. The single amplicon of expected size was amplified in all sunflower samples, in particular, primer pairs heli160f/heli160r and heli162f/heli162r generated 160 bp and 162 bp fragments, respectively (Figure 1, lanes 1–5, 6–10). While, the primer pairs heli177f/heli177r and heli188f/heli188r gave 177 bp and 188 bp amplicons, respectively (Figure 1, lanes 11–15, 16–19). However, PCR bands showed different intensities. Thus, the most intensive amplicons of heli-188 and heli-162 were identified as the best DNA markers for sunflower detection. In addition, no amplified products were seen for all maize and soybean samples in each PCR (Figure 1, lanes 3–4, 8–9, 13–14, 18–19) indicating high specificity of these PCR assays for sunflower identification. Moreover, the absence of any amplification signal in water-negative controls confirmed the absence of contaminating DNA (Figure 1, lanes 5, 10, 15). Therefore, results indicated that primer pairs heli188f/heli188r, heli160f/heli160r, and heli162f/heli162r were suitable for sensitive and specific detection of sunflower and they were applied in the following PCR experiments on sunflower oils.

### 3.2. Selection of Efficient DNA Extraction Methods for Sunflower Seeds and Oils

**Figure 2:**
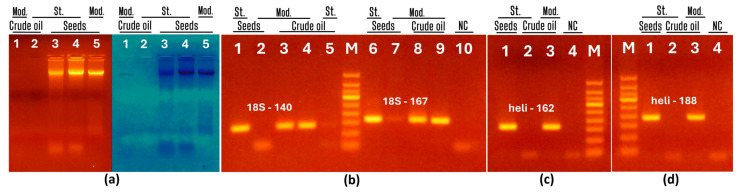
Comparison of CTAB protocols for DNA extraction from sunflower seeds and oils: (**a**) Genomic DNAs from crude oil (lanes 1–2) and seeds (lanes 3–5) extracted by modified CTAB (lanes 1, 5) and standard CTAB (lanes 2–4); (**b**–**d**) PCR amplification using primers 18S-140f/18S-140r (**b**, lanes 1–5), 18S-167f 18S-167r (**b**, lanes 6–10); heli162f/heli162r (**c**) and heli188f/heli188r (**d**). Samples: DNA from seeds (**b**, lanes 1–2, 6–7, **c**,**d**. lane 1) and crude oil (**b**, lanes 3–5, 8–9; **c**,**d**. 2–3) extracted by standard CTAB (**b**, lanes 1, 5, 6; **c**,**d**, 1–2) and modified CTAB (**b**, lanes 2–4, 7–9; **c**,**d**. lanes 3–4). Water-NC-negative control (**b**, lane 10, **c**,**d**, lane 4). M. Molecular weight marker (Qiagen GelPilot 100 bp ladder). Short notes: St.—standard CTAB; Mod.—modified CTAB.

The present work examined two variations of the cetyltrimethylammonium bromide (CTAB)–based method for isolating genomic DNA from sunflower seeds and oils. DNAs were extracted from 50 mL of pre-concentrated crude oil. The integrity of DNAs was assessed by agarose gel electrophoresis (Figure 2a). Intense bands of total genomic DNA were observed in both standard and modified CTAB-extracted seed samples (Figure 2a, lanes 3–5). Moreover, after image processing with Photoshop, a faintly visible DNA band appeared only in the modified CTAB-extracted oil sample (Figure 2a, right image, lane 1).

Amplifiability of genomic DNAs was assessed using two PCR systems targeting the eukaryotic 18S ribosomal RNA gene (Figure 2b) and two PCR systems targeting the sunflower *helianthinin* gene (Figure 2c,d). The expected amplicons were generated in seed samples extracted by the standard CTAB method (Figure 2b, lanes 1, 6, Figure 2c,d, lane 1). However, there was no amplified product in seed samples extracted with the modified CTAB protocol (Figure 2b, lanes 2, 7) despite the presence of whole genomic DNA (Figure 1, lane 5). This suggested the existence of PCR inhibitors in the seed DNA samples extracted with the modified CTAB protocol.

All four PCRs showed similar results for crude oil DNAs. In particular, the DNAs extracted by the modified CTAB method yielded amplicons of the expected size (Figure 2b, lanes 3–4, 8–9, Figure 2c,d, lane 3). However, crude oil DNAs obtained by the standard CTAB method did not generate a PCR product (Figure 2b, lane 5, Figure 2c,d, lane 2). The absence of amplified products in the negative control indicates the high purity of the PCR experiments (Figure 2b, lane 10, Figure 2c,d, lane 4). Therefore, the results revealed that the standard CTAB method is suitable for obtaining amplifiable genomic DNA from sunflower seeds and the modified CTAB method is suitable for obtaining amplifiable genomic DNA from sunflower oil.

**Figure 3:**
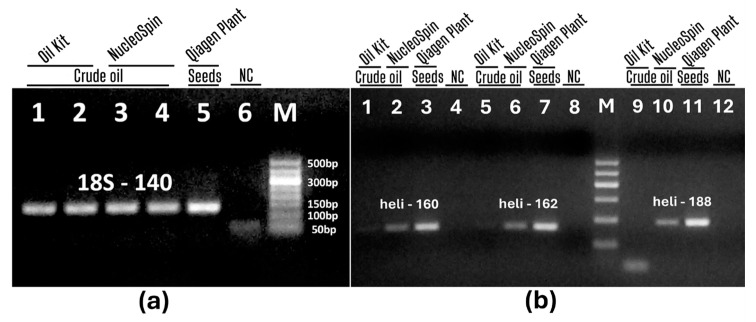
Comparison of Oil kit and NucleoSpin for DNA extraction from sunflower oil. PCR amplification using primer pairs 18S-140f/18S-140r (**a**, lanes 1–6); heli160f/heli160r (**b**, lanes 1–4); heli162f/heli162r (**b**, lanes 5–8) and heli188f/heli188r (**b**, lanes 9–12). Samples: DNAs from crude oil (**a**, lanes 1–4, **b**. lanes 1–2, 5–6, 9–10) and seeds (**a**, lane 5; **b**, lanes 3, 7, 11) extracted by Oil kit (**a**, lanes 1–2, **b**, 1, 5, 9), by NucleoSpin (**a**, lanes 3–4; **b**. 2, 6, 10) and by Qiagen plant (**a**, lane 5; **b**, lanes 3, 7, 11). Water-NC-negative control (**a**, lane 6, **b**. lanes 4, 8, 12). M. Molecular weight markers: Qiagen GelPilot 50 bp ladder (**a**) and Qiagen GelPilot 100 bp ladder (**b**).

Two DNA extraction kits (Oil kit and NucleoSpin) were evaluated to extract amplifiable genomic DNA from crude sunflower oil. 25 mL crude oil samples were pre-concentrated using protocol 1. The amplification ability of the DNAs was evaluated using eukaryotic and species-specific PCR systems, and the results are shown in Figure 3.

PCR with primers 18S-140f/18S-140r produced a single amplicon of the expected size and almost equal intensity for all oil samples extracted with both the oil kit and NucleoSpin (Figure 3a, lanes 1–4). However, PCR primers heli160f/heli160r, heli162f/heli162r, and heli188f/heli188r produced the expected PCR bands in the NucleoSpin samples (Figure 3b, lanes 2, 6, 10), but no visible PCR bands were detected in the Oil kit-derived samples (Figure 3b, lanes 1, 5, 9). The presence of amplified 18S-140 amplicons and the absence of heli-160, heli-162, and heli-188 amplicons in the oil kit samples is due to the higher efficiency of the 18S-140 PCR system compared to the helianthinin-specific PCR systems. The results indicate that both the oil kit and NucleoSpin can produce amplifiable DNA from sunflower oil, but NucleoSpin is more efficient because only its extracts allow detection of sunflower in crude oil samples.

In addition, seed DNAs extracted with the Qiagen plant kit gave the expected intense amplicons in each PCR system (Figure 3a, lane 5, Figure 3b, lanes 3, 7, 11). This indicates the suitability of the Qiagen plant kit to isolate sufficient amounts of amplifiable genomic DNA from sunflower seeds.

**Figure 4:**
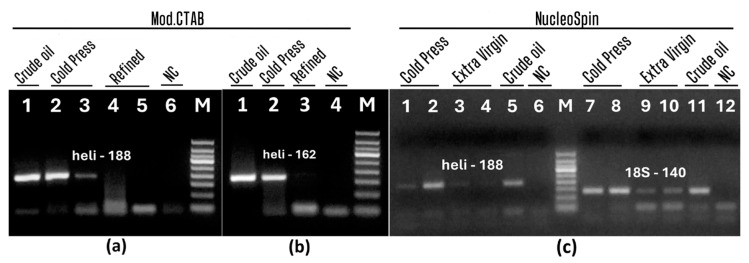
PCR testing of various pre-concentrated sunflower oils using primers: heli188f/heli188r (**a**, lanes 1–6; **c**, lanes 1–6); heli162f/heli162r (**b**, lanes 1–4); 18S-140f/18S-140r (**c**, lanes 7–12). Samples: crude (**a**, lane 1, **b**, lane 1, **c**, lanes 5, 11); cold press (**a**, lane 2–3, **b**. lane 2, **c**, lanes 1–2. 7–8); extra virgin (**c**, lanes 3–4, 9–10), refined (**a**, lane 4–5, **b**. 3) extracted by modified CTAB (**a**, lane 1–5, **b**, 1–3); NucleoSpin (**c**, lanes 1–5, 7–11). Water-NC-negative control (**a**, lane 6, **b**. lane 4, **c**, lanes 6, 12). M. Molecular weight markers: Qiagen GelPilot 50 bp ladder.

Furthermore, selected effective methods (modified CTAB and NucleoSpin) were tested to isolate sufficient amounts of amplifiable DNA from other types of oils, such as cold press, extra virgin, and refined sunflower oils. In order to determine the appropriate preconcentration protocol for efficient extraction, both preconcentration protocol 1 (Figure 4a,b) and protocol 2 (Figure 4c) were used. 12 mL, 24 mL, and 48 mL of all types of oils, namely crude, cold pressed, extra virgin, and refined oils were tested to select the optimal volume for each oil. Moreover, 300 mL of refined oil was concentrated by pre-concentration protocol 1. The oil extracts were examined by PCR with primers specific for *helianthinin* gene and the 18S RNA gene.

As shown in Figure 4, gel electrophoresis revealed the expected PCR products in all crude and cold-pressed oils (Figure 4a, lanes 1–3, Figure 4b, lanes 1–2, Figure 4c, lanes 1–2, 7–8, 5, 11) preconcentrated by both protocols and extracted by both modified CTAB and NucleoSpin methods. This suggested the suitability of both pre-concentration approaches as well as the modified CTAB and NucleoSpin methods for obtaining amplifiable genomic DNA from oil. This suggested suitability of the both preconcentration approaches and both modified CTAB and NucleoSpin methods to obtain amplifiable genomic DNA from oil. However, no amplicons were obtained after PCRs using *helianthinin* gene-specific primers in all extra virgin and refined oils extracted by both the modified CTAB and NucleoSpin methods (Figure 4a, lanes 4–5, Figure 4b, lane 3, Figure 4c, lanes 3–4). 140 bp PCR bands were visible in all oil samples (crude, extra virgin, and refined) as expected, confirming the amplifiability of all DNA samples (Figure 4c, lanes 7–12). While, extra virgin oil samples yielded amplicons of lower intensity (Figure 4c, lanes 9–10), indicating the presence of lower amounts of genomic DNA in extra virgin oil than cold pressed oil. This was confirmed by helianthinin-specific PCR. In addition, PCR bands of similar intensity were detected in DNA samples obtained by both the modified CTAB and NucleoSpin methods from pre-concentrated 12 mL of crude oil and 48 mL of cold-pressed oil (Figure 4a, lanes 1–2, Figure 4b, lanes 1–2, Figure 4c, lanes 1–2, 5, 7–8, 11). This indicated that the crude oil sample contained more genomic DNA than the cold-pressed oil, and the cold-pressed oil sample had more DNA than the extra-virgin or refined oil samples. This suggests that despite the amplifiability of DNAs from all crude, cold-pressed, extra virgin, and refined oils, they have different PCR efficiencies due to different amounts of DNA.

### 3.3. Development of Nested and Double PCRs for Sunflower Detection in Edible Oils

The study described above suggested that improvement of PCR sensitivity was needed to detect sunflowers in extra virgin and refined oils. To this purpose, double and nested PCR approaches were applied. The primer pairs heli188f/heli188r and heli162f/heli162r were suitable for the nested PCR system because the amplicon heli-162 is present inside the amplicon heli-188, moreover, forward primers heli188f and heli162f contain a common sequence as an important part. In addition, a double PCR was performed, where heli188f/heli188r-primers were used both in the first and second PCR. Initially, DNAs from pre-concentrated 12 mL oil samples were tested. Both nested and double PCRs produced very intense PCR bands, so the volume of oil samples was reduced in subsequent experiments. Finally, 700 µL of oil was sampled without pre-concentration and subjected to DNA extraction by four methods such as NucleoSpin, Qiagen Food, olive oil kit, and CTAB methods. The resulting genomic DNAs were evaluated with a Nanodrop One spectrophotometer.

Table 2 presents the concentration and purity values for the DNAs obtained from 700 μL of the oils by the four extraction protocols. DNA concentrations and purity were estimated by measuring the UV absorbance at 260 nm (A_260_) and the ratios of the absorbance A_260_/A_280_ and A_260_/A_230_, respectively. Mean and standard deviation were calculated for the data obtained from DNA concentration and purity. Values were then expressed as Mean ± SD.

Statistical analyses performed by ANOVA indicate that oil type does not significantly influence DNA concentration and purity (260/280 and 260/230). However, the DNA extraction method affects both the DNA concentration and the 260/230 purity values, while it does not affect the 260/280 purity values. Notably, all three DNA extraction kits such as NucleoSpin, Qiagen Food, and Oil Kit combined into one group which is different from the other group of the CTAB method. Three homogeneous groups were identified based on purity values of 260/230. The Qiagen Food kit and the modified CTAB method were combined into one group, although the NucleSspin and Oil Kit were identified as separate distinct groups.

Comparison of DNA concentrations and yields exhibited differences between extraction methods, although the types of oils did not show significant differences. The modified CTAB enabled higher DNA concentration (mean 5.86 ng/μL), followed by NucleoSpin (mean 3.28 ng/μL) and oil kit (mean 2.56 ng/μL). The Qiagen food kit produced extracts at lower concentrations (mean 1.40 ng/μL). However, Nucleospin (mean 468.6 ng/mL oil) and modified CTAB (mean 418.5 ng/mL oil) gave higher DNA yields, while the Qiagen food and oil kit yielded relatively less DNA, averaging 300.00 ng and 256 ng per mL of oil, respectively.

The ratios of the absorbance A_260_/A_280_ and A_260_/A_230_ were used to estimate the purity of DNAs and the amount of organic contaminants, such as proteins, phenols, and other aromatic compounds. Higher values for mean A_260_/A_280_ ratios were obtained with modified CTAB (2.21), and oil kit (1.95), but the other two methods showed lower values such as 1.68 for NucleoSpin and 1.54 for Qiagen Food. The results suggested the absence of protein contamination in most DNA samples because the A_260_/A_280_ ratio was greater than 1.8. Only three NucleoSpin and two Qiagen Food samples showed A_260_/A_280_ ≤ 1.7, indicating the presence of proteins in these samples. Different results were exhibited for ratios A_260_/A_230_, namely, NucleoSpin showed the highest values (0.54) followed by oil kit (0.43). Lower values of 0.25 and 0.22 were obtained for Qiagen Food and modified CTAB, respectively. The results obtained indicate the presence of phenols and other compounds as all ratios A_260_/A_230_ are lower than 2.0.

**Figure 5:**
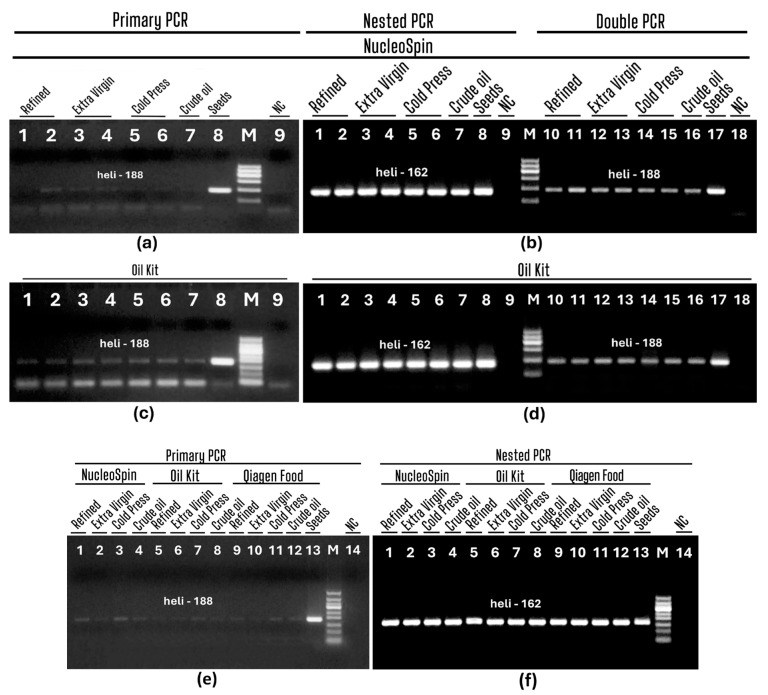
Primary (**a**,**c**,**e**), Nested (**b**,**d**, lanes 1–9; **f**, lanes 1–14) and Double (**b**,**d**, lanes 10–18) PCRs using primers heli188f/heli188r (**a**,**c**,**e**, and **b**,**d**, lanes 10–18) and heli162f/heli162r (**b**,**d**, lanes 1–9, **f**, lanes 1–14) of sunflower DNA extracted from oils by NucleoSpin (**a**, lanes 1–9, **b**, lanes 1–18, **e**,**f**, lanes 1–4), oil kit (**c**, lanes 1–9, **d**, lanes 1–18, **e**,**f**, lanes 5–8); Qiagen Food (**e**,**f**, lanes 9–12). Samples: refined (**a**,**c**, lanes1–2; **b**,**d**, lanes 1–2, 10–11; **e**,**f**, lanes 1, 5, 9); extra virgin (**a**,**c**, lanes 3–4, **b**,**d**, lanes 3–4, 12–13; **e**,**f**, lanes 2, 6, 10); cold press (**a**,**c**, lanes 5–6, **b**,**d**, lanes 5–6, 14–15; **e**,**f**, lanes 3, 7, 11); crude (**a**,**c**, lane 7, **b**,**d**, lanes 7, 16; **e**,**f**, lanes 4, 8, 12); seeds (**a**,**c**, lane 8; **b**,**d**, lanes 8, 17; **e**,**f**, lane 13); water-NC-negative control (**a**,**c**, lane 9; **b**,**d**, lanes 9, 18; **e**,**f**, lane 14); M. Molecular weight markers: Qiagen GelPilot 100 bp ladder (**a**,**b**,**d**) and Qiagen GelPilot 50 bp ladder (**c**,**e**,**f**).

To evaluate the sensitivity of sunflower detection, the PCR products of the uniplex, nested, and double PCRs were assessed by agarose gel electrophoresis and are presented in Figure 5. DNAs extracted with the NucleoSpin and olive oil kits were tested by both double and nested PCR (Figure 5b–d) to select the best approach. As shown in Figure 5a,b, uniplex PCRs with primers heli188f/heli188r produced slightly visible PCR bands for all oils (refined, extra virgin, cold pressed, and crude) extracted by both NucleoSpin (a, lanes 1–9; b, lanes 1–18), as well as with an oil kit (c, lanes 1–9, d, lanes 1–18). Double PCRs with primers heli188f/heli188r generated well-visible 188 bp amplicons for DNAs obtained with the NucleoSpin and olive oil kits (Figure 5b,d, lanes 10–18). However nested PCRs with primers heli162f/heli162r produced very intense PCR bands for both extraction kits (Figure 5b,d, lanes 1–9). Therefore, the obtained results revealed nested PCR as the best approach for the detection of sunflower in oil.

Subsequently, nested PCR was performed using the DNAs extracted by the Qiagen Food kit. The intensity of PCR bands is sensitive to differences in agarose gel electrophoresis. Thus, the PCR products of NucleoSpin, oil kit, and Qiagen Food-derived DNAs were run on the same agarose gel (Figure 5e,f) to compare these three extraction methods. Weak PCR bands were observed after uniplex PCRs for each tested oil extracted by all methods (Figure 5e, lanes 1–12). However, strong PCR bands obtained by nested PCR in all oil samples indicated the same efficiency of all extraction methods (Figure 5f, lanes 1–12). In addition, sunflower seed DNA generated exceptionally strong amplicons not only by nested PCR but also by uniplex and dual PCR, confirming the sufficient efficiency of these PCR systems (Figure 5a,c, lane 8; Figure 5b,d, lanes 8, 17; Figure 5e,f, lane 13).

**Figure 6:**
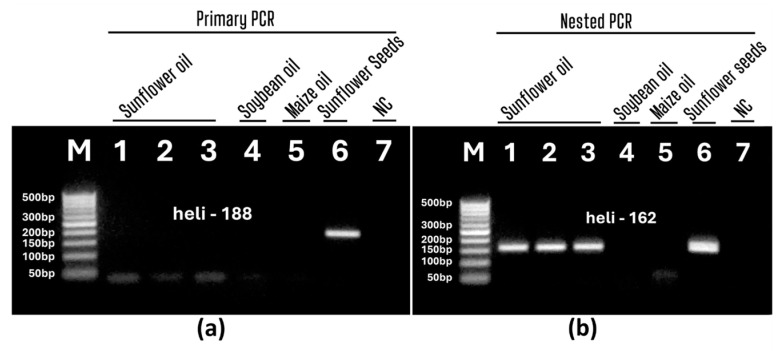
Primary (**a**) and nested (**b**) PCRs with primers heli188f/heli188r (**a**) and heli162f/heli162r (**b**) of DNAs from different oils. Samples: sunflower oils (**a**,**b**, lanes 1–3), soybean oil (**a**,**b**, lane 4) and maize oil (**a**,**b**, lane 5), sunflower seeds (**a**,**b**, lane 6); water-NC-negative control (**a**,**b**, lane 7, **b**). Molecular weight marker: Qiagen GelPilot 50 bp ladder (**a**,**b**).

To verify the specificity of the detection method, soybean and maize oils were tested by the developed nested PCR (Figure 6). The primary PCR produced no products for the sunflower, soybean, and maize oil samples (Figure 6a, lanes 1–5), while the expected 188 bp amplicon appeared in the sunflower seed DNA sample (Figure 6a, lane 6). However, nested PCR produced the expected 162 bp amplicons in the sunflower oil and seed samples (Figure 6b, lines 1–3, 6), while no product appeared in the soybean and maize oil samples (Figure 6b, lines 4–5). Therefore, the results showed the high specificity of the developed nested PCR in the identification of sunflower in oils. It should be noted that no PCR product was formed in the negative water control in any of the PCR experiments, which indicates the high purity and specificity of the experiments.

### 3.4. Tracing of Sunflower in UCO

**Figure 7:**
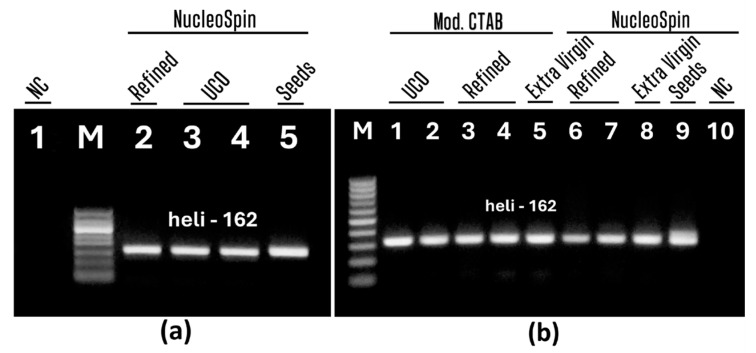
Second PCRs using primers heli162f/heli162r of DNAs from sunflower oils and UCO extracted by NucleoSpin kit (**a**, lanes 2–5, **b**, lanes 6–8) and modified CTAB (**b**, lanes 1–5). Samples: refined oil (**a**, lanes 2, **b**. lanes 3–4, 6–7); extra virgin oil (**b**, lanes 5, 8); UCO (**a**, lanes 3–4, **b**, lanes 1–2); seeds (**a**, lane 5, **b**, lane 9), water-NC-negative control (**a**, lane 1, **b**, lane 10). Molecular weight marker: Qiagen GelPilot 50 bp ladder (**a**,**b**).

The developed nested PCR was applied to trace sunflower in used cooking oil. Figure 7 shows agarose gel electrophoresis of nested PCR products. Both NucleoSpin and modified CTAB-derived oil extracts were analyzed. Refined and extra virgin oils were tested with UCO to compare method sensitivity for these oils. Sunflower seed DNA was tested to verify the efficiency of PCR experiments.

Single amplicons of 162 bp size were generated by nested PCR for all oil samples, including UCO (a, lanes 3–4, b, lanes 1–2), refined (a, lanes 2; b, lanes 3–4, 6–7) and extra virgin (b, lanes 5, 8) oils extracted by both the NucleoSpin (a, lanes 2–5; b, lanes 6–8) and modified CTAB (b, lanes 1–5) methods. Amplicons had similar and high intensities (Figure 7a lanes 2–4, Figure 7b lanes 1–8) in all oil samples, indicating the suitability of the nested PCR method for testing sunflowers in UCO. The highest intensity PCR product was produced in the seed sample, as expected. The absence of amplified product in the water negative control (a, lane 1; b, lane 10) indicated the purity of the experiments.

## 4. Discussion

The detection of sunflower in oils is important for both food and biodiesel production. In this study, PCR-based technology was selected as a widely used approach to efficiently and reliably detect ingredients in processed foods. The analytical procedure for PCR detection consists of several steps: sample preparation, genomic DNA extraction, and PCR amplification. Effective performance of each step is an important prerequisite for successful PCR detection. Sufficient amounts of amplifiable genomic DNA and efficient PCR systems are important aspects of PCR sensitivity. In the present study, a comprehensive investigation and optimization of the critical factors of PCR detection was carried out to achieve highly sensitive identification of sunflowers in strongly processed oils. The following factors were examined: oil type and amount, treatment forms, DNA extraction method, target gene copy number, PCR primers, amplicon size, and PCR approach.

In this work, four types of sunflower oil (crude, cold pressed, extra virgin, and refined) as well as UCO were investigated to determine the influence of oil type, characteristics, and forms of processing that affect PCR detection. The crude, cold-pressed, and extra virgin oils were produced by mechanical or chemical processing. However, refined oil has undergone high-temperature treatment. UCO was obtained from refined oils after thermal processing applied in cooking. In addition, UCO has undergone mechanical filtration at a biodiesel company. It should be noted that the oils were produced by different companies and had different characteristics. In particular, crude, cold-pressed, and used cooking oils were produced locally by different small manufacturers and were provided very quickly after production, they had characteristic odor and taste. In addition, visible precipitation was observed after storage. The extra virgin and refined oils were products of large industrial enterprises, and they were imported from other countries. Thus, they had to undergo more rigorous processing to obtain a final product with acceptable organoleptic properties and maintain quality for a long time. After storage, there was no smell or visible pallet in their bottles.

Significant differences between the PCR results of cold-pressed and refined oils were previously reported in other works and were explained by severe processing during oil refining [30,31,33]. In the present study, an unexpected significant difference was observed between the PCR results of cold-pressed and extra virgin oils. In particular, DNAs from extra-virgin and refined oils showed similar PCR efficiency, but it was much lower than that of cold-pressed oil DNAs. This indicated that despite non-thermal processing of extra virgin oil it has undergone additional cleaning and processing in industrial production, as it was intended for transportation and long-term storage. While cold-pressed oils were relatively poorly processed in small local enterprises. Thus, the PCR efficiency of oil DNA depends on both the type of oil and the form of processing used in production. Therefore, they can be considered critical factors affecting oil PCR detection.

Proper sampling and DNA extraction are considered obstacles for PCR analysis of oil [12,31,48]. Selecting the appropriate volume of oil is an important factor for correct sampling. In this study, different volumes (between 500 µL and 300 mL) of four types of sunflower oil (crude, cold pressed, extra virgin, and refined) were investigated to select the correct volume for successful PCR amplification. Electrophoretic analysis of uniplex PCR products of pre-concentrated oils showed that despite the amplifiability of all DNAs of the oils, the detection efficiency of sunflower by uniplex PCR was different due to the different amounts of DNA in the oil extracts. In particular, the crude oil sample contained more genomic DNA than the cold-pressed oil, and the cold-pressed oil sample had more DNA than the extra virgin or refined oil samples. Moreover, similar PCR efficiency was observed in pre-concentrated 12 mL crude oil and 48 mL cold-pressed oil samples, suggesting that more processing was used on cold-pressed oil than on crude oil due to different production by different companies. In addition, PCR detection of sunflower failed even when the volume of refined oil was increased to 300 mL.

A sufficient amount of amplifiable genomic DNA is a crucial prerequisite for successful PCR detection. The harsh processing in oil production affects the integrity of DNA and leads to high degradation of DNA fragments [10,12,30,31,33]. Due to the low amount of DNA in the oils, pre-concentration [31] or DNA enrichment [46,48,49,50] procedures were often applied to extract DNA from large volume (10–500 mL) oil samples. Therefore, available extraction methods are often very laborious, long, and expensive, and may fail to generate amplifiable DNA. Two pre-concentration protocols were successfully used in the present work. It should be emphasized that the pre-concentration protocol 2 is a very simple, rapid, and inexpensive procedure developed in this study. It has the advantage of using centrifugation in a 2 mL mini-centrifuge tube for 20 min at room temperature and is convenient for concentrating relatively small to medium volume (1–48 mL) samples. While pre-concentration 1 [31] requires a large refrigerated centrifuge with larger tubes (≥25 mL) and is more suitable for the concentration of large-volume samples.

This study identified the DNA extraction method as a critical factor for PCR detection of oil based on differences in the amplification of genomic DNAs isolated by three DNA extraction kits and two CTAB protocols. Despite the availability of commercial kits, various modifications of CTAB-based methods are still widely used to extract DNA from plant foods, including oils [30,35,38,46,48,49,50,51]. This is due to the availability of additional optimization of CTAB methods and the relatively low cost compared to expensive ready-made DNA isolation kits. Sunflower contains high concentrations of polyphenols, polysaccharides, and tannins that prevent amplifiable DNA isolation and are considered as PCR inhibitors. Earlier works described optimized procedures using the QIAamp DNA Stool Mini Kit (Qiagen) [10] and modified CTAB [30] for DNA extraction from 15 mL and 30 mL sunflower oil without sample pretreatment.

Two (standard and our modified) CTAB protocols were tested for DNA extraction from sunflower seeds and crude oil. To increase the amount of DNA isolated from the oil, a modified CTAB protocol was developed that lacked the final purification steps of the standard CTAB method. The results showed that amplifiable DNA was obtained from the seeds only by the standard CTAB method and from the oil only by the modified protocol. This suggests that vigorous purification by the standard CTAB method is important for seed DNA because its extract is rich in PCR inhibitors. A short modified protocol supports the isolation of more DNA from oil samples, in which the main challenge is the small amount of DNA. It is important to note that our modified CTAB protocol ensures fast, cheap, and easy extraction of oil DNA and facilitates oil PCR detection. As far as we know Figure 2a represents a unique picture of a visible band of oil DNA that has not yet been previously obtained.

Two DNA extraction kits, namely NucleoSpin and Qiagen Food, were selected for this study because they have previously been successfully used for other oils such as soybean, canola, cottonseed, and peanut oils [12,31,52]. In addition, the olive oil kit was chosen based on its specificity for oils. Examination of commercial kits of DNA extraction revealed Nucleospin, Qiagen food, and Oil kits suitable for sunflower oil as well as Qiagen plant kits useful for sunflower seeds. These kits were applied to sunflower oil for the first time. Our results are consistent with previous findings of the effectiveness of the traditional CTAB method in removing polyphenols, but its inability to extract DNA from oil. In addition, earlier reports on the effectiveness of the NucleoSpin and Qiagen Food kits in extracting amplifying DNA from oils were confirmed [12,31,52].

This study is the first to present spectrophotometric data for sunflower oils. The results coincide with previous reports about the dependence of DNA yields and purity on the extraction method used for oil DNA isolation [12,31,48,50]. The low ratios A260/A230 between 0.18 and 0.62 coincide with previous data [12] and indicate remaining organic compounds such as polysaccharides, phenols, etc. in the oil DNA extracts. Comparison and interpretation of electrophoretic and spectrophotometric results obtained for DNAs from 700 µL of oils suggested that all DNAs extracts were amplifiable and generated expected amplicons by both the double and nested PCRs despite low DNA concentration (about 1.3 ng/µL) and low purity (ratios A260/A280 about 1.1 and A260/A230 about 0.18). Our outcomes confirm previous findings that the contaminants in the oil DNA extracts may not inhibit the PCR [12,31].

The PCR approach and the number of copies of the target DNA largely determine the sensitivity of the PCR assay. In this study, a nested PCR approach was chosen as a highly sensitive method for DNA detection. Nested and double PCR allows for increased PCR sensitivity by direct re-amplification of the product from the primary PCR with a second PCR [53]. This technique is considered a promising tool for the analysis of processed foods. Its application to trace virgin olive oil [54], as well as transgenic soybean, corn, and canola oils [38] allowed to reduce the oil sample volume to 200 μL and 2 mL, respectively.

The PCR approach for species identification is based on the detection of a specific DNA sequence characteristic of that species. Debode et al. [33] reported that the use of species-specific sequences of chloroplastic DNA and ribosomal RNA genes as high copy number targets gave better results than low cellular copy number targets in PCR detection of oils. Notably, multicopy targets provide a better signal, but they are not suitable for quantitative purposes because the copy number can vary in the cells [33]. Previous studies used PCRs targeting sunflower-specific sequences of multicopy rDNA and plastid DNA to detect sunflower in foods [40,41] and in oils [10], respectively.

In this study, two eukaryote-specific PCR systems producing 140 bp [45] and 167 bp fragments [46] of the multicopy18S RNA gene were used to check the amplifiability of oil DNAs. It should be noted that the comparative analysis of 18S-140 and 18S-167 PCR products in concentrated oils contributed to the development of pre-concentration II and modified CTAB methods. These PCR systems were able to generate amplified products by uniplex PCR, even in DNAs from 700 μL of oils. Our results confirmed previous reports on the successful use of primers 18S-140f/18S-140r [45] to amplify DNAs from processed foods and primers 18S-167f/18S-167r to amplify DNAs from maize and soybean oils [46]. This study revealed target gene copy number as a critical factor for oil PCR detection based on the comparison of the results for PCRs targeting the multicopy18S RNA gene and the low-copy *helianthinin* gene.

Commonly, species-specific protein genes are utilized as low-copy number targets in PCR for reliable identification and quantification of species DNA. In this study, the *helianthinin* gene was chosen as a target gene to develop PCR systems for sunflower detection because of the available data on the sunflower species-specificity of Helianthinins proteins [47]. Moreover, a PCR assay targeting the 60 bp sequence of the *helianthinin* gene was developed by Hernandez et al. [39] and then used to identify sunflower in edible oils by He et al. [30]. In the present work, three uniplex PCR systems were developed for the detection of sunflowers using newly designed primer pairs. Moreover, two pairs of them, producing amplicons of 188 bp and 162 bp, were successfully used in nested and double PCR systems.

Examination of several primer pairs as well as uniplex, double and nested PCRs made it possible to identify PCR primers and approaches as important factors in PCR analysis of oil. The use of both double and nested PCRs dramatically changed the sensitivity of sunflower detection in oils. Uniplex PCRs allowed the detection of sunflower only in concentrated 12 mL crude and 48 mL cold-pressed oil samples, but these methods failed to analyze extra virgin and refined oils. However, double and nested PCRs enabled sunflower tracking in 700 µL of all studied oils extracted by NucleoSpin, oil kit, Qiagen Food, or modified CTAB methods. It should be emphasized that nested PCR exhibited a higher sensitivity than double PCR. The absence of amplified products in DNAs of maize and soybean seeds and oils as well as in water-negative controls showed high specificity and purity of PCR assays. Thus, the application of the nested PCR method resulted in efficient and reliable detection of sunflower in edible and used cooking oils. The ability to detect sunflowers in 700 μL of refined and used cooking oils improved the sensitivity of previous methods that detected sunflowers in 15 mL and 30 mL of edible oil [10,30].

Further experiments are needed to validate the developed technology for detecting sunflowers in blended oils. It would also be possible to develop a similar technology to detect other oilseeds in oils.

Noteworthy, the use of modified CTAB in combination with nested PCR provides cheap, fast, and efficient tracking of sunflowers in oils. Compared to existing methods of oil authentication, the main advantages of the technology developed in this study are higher sensitivity, less amount of oil samples, and a simple DNA extraction method. Furthermore, this is the first report of sunflower detection in UCO, which may be useful for both food and biodiesel production.

## 5. Conclusions

This study demonstrated that the critical factors for oil PCR analysis are oil type, amount, and treatment forms, as well as the extraction method, target gene copy number, and PCR approach. Although oil processing severely affects DNA integrity and amount proper choice of these factors may lead to effective oil authentication.

The highly sensitive, simple, and rapid technology was developed for the reliable detection of sunflower in edible and used cooking oils. Nested PCR using newly designed primers dramatically increased the sensitivity of the detection and enabled the identification of sunflower in even 700 µL of oil. Tracking of sunflowers in the used cooking oils can be considered a notable achievement due to the absence of published reports concerning the authentication of UCO.

It was demonstrated that NucleoSpin, Qiagen food, and Oil kits as well as modified CTAB method allow the extraction of amplifiable genomic DNA from sunflower oils. Combining each of these extraction methods with the developed nested PCR enabled efficient and accurate detection of trace amounts of sunflower in oil. Consequently, the technology presented herein may be successfully applied for sunflower tracking in edible and used cooking oils during food and biodiesel production.

The present work meets critical challenges of sustainable food and biofuel systems, such as food security, food waste utilization, and eco-friendly fuel production. These challenges are closely related to sunflower oils, which are widely used in food preparation and result in waste cooking oils. The use of WCO in biodiesel production is an excellent example of using food waste and contributes to the sustainability of the food and biofuel industries.

## Figures and Tables

**Table 1:** Oligonucleotide primers used in PCR.

Primer	Sequence 5′→3′	Target Gene	Amplicon Size (bp)	Reference
heli160f	TCAACGCCCACAATCTTCTC	*helianthinin*	160	This study
heli160r	CTTCCTTGTTCATTGGCTCTCT			
heli162f	CTTCCCAGGCTGACTTTGTAA	*helianthinin*	162	This study
heli162r	GAAGATTGTGGGCGTTGATTG			
heli177f	CCTTCCTACGTCAACACCCC	*helianthinin*	177	This study
heli177r	TCATAGGTTCTGCGGCATCC			
heli188f	CCTTCCCAGGCTGACTTTGT	*helianthinin*	188	This study
heli188r	CTCAAGGCTCCCTCGGTTAC			
18S-140f	TCTGC-CCTATCAACTTTCGATGGTA	*18S rRNA*	140	[45]
18S-140r	AATTTGCGCGCCTGCTGCCTTCCTT			
18S-167f	GCAAGACCGAAACTCAAAGGA	*18S rRNA*	167	[46]
18S-167r	ACGACAGCCATGCAGCACC			

**Table 2:** DNA concentration and purity of the oil extracts obtained with NucleoSpin, Qiagen Food, oil kit, and modified CTAB methods.

Extraction Method	Oil Samples (0.7 mL)	DNA (ng/µL)	A_260/A280_	A_260/A230_
NucleoSpin	Crude	2.39 ± 0.76 ^a^	1.80 ± 0.90 ^a^	0.53 ± 0.09 ^a^
	Cold pressed	1.51 ± 0.64 ^a^	1.60 ± 0.50 ^a^	0.42 ± 0.07 ^a^
	Extra virgin	4.82 ± 2.07 ^a^	1.49 ± 0.07 ^a^	0.59 ± 0.04 ^a^
	Refined	4.25 ± 0.90 ^a^	1.42 ± 0.01 ^a^	0.62 ± 0.02 ^a^
	UCO	3.415 ± 0.70 ^a^	2.09 ± 0.05 ^a^	0.55 ± 0.07 ^a^
Qiagen Food kit	Crude	1.41 ± 0.02 ^a^	1.14 ± 0.04 ^a^	0.24 ± 0.01 ^c^
	Cold pressed	1.49 ± 0.55 ^a^	1.00 ± 0.34 ^a^	0.25 ± 0.03 ^c^
	Extra virgin	1.30 ± 0.14 ^a^	2.14 ± 1.58 ^a^	0.25 ± 0.02 ^c^
	Refined	1.40 ± 0.00 ^a^	1.87 ± 0.20 ^a^	0.25 ± 0.02 ^c^
Oil Kit	Crude	3.45 ± 0.10 ^a^	2.03 ± 0.31 ^a^	0.46 ± 0.00 ^b^
	Cold pressed	2.58 ± 0.55 ^a^	2.03 ± 0.21 ^a^	0.45 ± 0.05 ^b^
	Extra virgin	2.47 ± 1.75 ^a^	1.73 ± 0.17 ^a^	0.44 ± 0.16 ^b^
	Refined	1.72 ± 0.56 ^a^	2.02 ± 0.10 ^a^	0.36 ± 0.07 ^b^
Modified CTAB	Crude	5.15 ± 0.54 ^b^	2.32 ± 0.13 ^a^	0.24 ± 0.02 ^c^
	Cold pressed	5.41 ± 0.31 ^b^	2.62 ± 0.80 ^a^	0.25 ± 0.10 ^c^
	Extra virgin	6.58 ± 1.75 ^b^	2.11 ± 0.31 ^a^	0.20 ± 0.01 ^c^
	Refined	5.12 ± 0.71 ^b^	1.91 ± 0.01 ^a^	0.18 ± 0.01 ^c^
	UCO	7.05 ± 1.68 ^b^	2.09 ± 0.04 ^a^	0.24 ± 0.02 ^c^

Different superscript letters within the column indicate statistically significant differences between groups, as determined by Tukey’s Honestly Significant Difference (HSD) test (*p* < 0.05). Groups sharing the same letter are not significantly different from each other.

## Data Availability

All data generated and analyzed during this study are included in the present article. Further inquiries can be directed to the corresponding author.
